# PDGF-BB serum levels are decreased in adult onset Pompe patients

**DOI:** 10.1038/s41598-018-38025-0

**Published:** 2019-02-14

**Authors:** Esther Fernández-Simón, Ana Carrasco-Rozas, Eduard Gallardo, Sebastián Figueroa-Bonaparte, Izaskun Belmonte, Irene Pedrosa, Elena Montiel, Xavier Suárez-Calvet, Jorge Alonso-Pérez, Sonia Segovia, Claudia Nuñez-Peralta, Jaume Llauger, Mercedes Mayos, Isabel Illa, Spanish Pompe Study Group, Miguel Angel Barba-Romero, Joseba Barcena, María Rosario Carzorla, Carlota Creus, Jaume Coll-Cantí, Noemí de Luna, Manuel Díaz, Cristina Domínguez, Roberto Fernández-Torrón, María José García-Antelo, Josep María Grau, María Teresa Gómez-Caravaca, Juan Carlos León-Hernández, Adolfo López de Munáin, Francisco Antonio Martínez-García, Yolanda Morgado, Antonio Moreno, Germán Morís, Miguel Angel Muñoz-Blanco, Andres Nascimento, Carmen Paradas, José Luis Parajuá-Pozo, Luis Querol, Arturo Robledo-Strauss, Ricard Rojas-García, Íñigo Rojas-Marcos, Jose Antonio Salazar, Mercedes Usón, Jordi Díaz-Manera

**Affiliations:** 1 Neuromuscular Disorders Unit. Neurology Department Hospital de la Santa Creu i Sant Pau. Universitat Autònoma de Barcelona, Barcelona, Spain; 2 Centro de Investigación en Red en Enfermedades Raras (CIBERER), Barcelona, Spain; 3 Rehabilitation and physiotherapy department Hospital de la Santa Creu i Sant Pau. Universitat Autònoma de Barcelona, Barcelona, Spain; 4 Radiology department Hospital de la Santa Creu i Sant Pau. Universitat Autònoma de Barcelona, Barcelona, Spain; 5 Respiratory department. Hospital de la Santa Creu i Sant Pau. Universitat Autònoma de Barcelona, Barcelona, Spain; 6 Hospital General de Albacete, Albacete, Spain; 7 Hospital Universitario Cruces, Baracaldo, Spain; 8 Hospital Puerta de Hierro, Majadahonda, Spain; 9 Hospital Virgen de las Nieves, Granada, Spain; 10 Hospital Germans Tries i Pujol, Badalona, Spain; 11 Hospital de Cabueñes, Gijón, Spain; 12 Hospital 12 de Octubre, Madrid & Insituto de Investigación i+12, Madrid, Spain; 13 Hospital Universitario Donostia, Donostia, Spain; 14 Hospital Universitario A Coruña, A Coruña, Spain; 15 Hospital Clínic, Barcelona, Spain; 16 Hospital de Córdoba, Córdoba, Spain; 17 Hospital Universitario Nuestra Señora de la Candelaria, Tenerife, Spain; 18 Hospital Clínico Universitario Virgen de la Arrixaca, Murcia, Spain; 19 Hospital Universitario Virgen de Valme, Sevilla, Spain; 20 Hospital Universitario Morales Meseguer, Murcia, Spain; 21 Hospital Universitario de Asturias, Oviedo, Spain; 22 Hospital Gregorio Marañón, Madrid, Spain; 23 Hospital Sant Joan de Déu, Barcelona, Spain; 24 Hospital Virgen del Rocío, Sevilla, Spain; 25 Hospital de Can Mises, Ibiza, Spain; 26 Hospital Juan Ramón Jiménez, Huelva, Spain; 27 Hospital Virgen de Macarena, Sevilla, Spain; 28 Hospital Regional Universitario de Málaga, Málaga, Spain; 29 Hospital de Son Llátzer, Palma de Mallorca, Mallorca, Spain

## Abstract

Adult onset Pompe disease is a genetic disorder characterized by slowly progressive skeletal and respiratory muscle weakness. Symptomatic patients are treated with enzymatic replacement therapy with human recombinant alfa glucosidase. Motor functional tests and spirometry are commonly used to follow patients up. However, a serological biomarker that correlates with the progression of the disease could improve follow-up. We studied serum concentrations of TGFβ, PDGF-BB, PDGF-AA and CTGF growth factors in 37 adult onset Pompe patients and 45 controls. Moreover, all patients performed several muscle function tests, conventional spirometry, and quantitative muscle MRI using 3-point Dixon. We observed a statistically significant change in the serum concentration of each growth factor in patients compared to controls. However, only PDGF-BB levels were able to differentiate between asymptomatic and symptomatic patients, suggesting its potential role in the follow-up of asymptomatic patients. Moreover, our results point to a dysregulation of muscle regeneration as an additional pathomechanism of Pompe disease.

## Introduction

Pompe disease is an autosomal recessive disorder produced by mutations in the *GAA* gene, which codifies the enzyme acid alpha-glucosidase^1^. This enzyme metabolizes glycogen to glucose inside the lysosomes of the cells. A lack of it leads to an accumulation of glycogen in body tissues such as liver, neurons, smooth, skeletal or cardiac muscle^2^. Pompe disease has a wide clinical spectrum ranging from the classical infantile disease (IOPD) to late onset phenotype. IOPD patients develop a quickly progressive disease characterized by generalized muscle weakness and hypertrophic cardiomyopathy, leading to death in the first year of life if left untreated^3^. Enzymatic replacement therapy with alfa-glucosidase (ERT) is started as early as possible, because IOPD is life-threatening disorder and ERT clearly change patients’ clinical condition^4,5^.

In contrast, adult onset Pompe patients (AOPD) can have heterogeneous clinical presentations, ranging from isolated hyperckemia to weakness involving the respiratory, axial, pelvic and scapular girdle muscles^6,7^. Natural history studies suggest that muscle weakness in AOPD progresses very slowly. In some cases, patients develop subtle muscle symptoms, such as postural abnormalities or a change in their walking pattern, that do not influence general motor function and can therefore delay medical consultation^8,9^. Unlike the procedure for IOPD, guidelines recommend starting ERT in AOPD only if muscle or respiratory weakness is detected in clinical examination^10^. In fact, asymptomatic AOPD patients are currently followed up using different muscle function tests to try to identify changes in motor performance that could lead to the initiation of treatment^11^. However, it is possible that the process of muscle degeneration and fibro-fatty substitution, which is irreversible, could have started without yet producing significant changes in motor functional tests^12^. Moreover, it is not certain that common muscle function tests, such as the 6 minute walking test (6MWT), are precise enough to detect slight changes in motor performance^13^. For these reasons, having a serum growth factor able to identify patients in which fibro-fatty substitution has begun, would be of great utility^14,15^.

The main aim of our research was to study the serum concentration of a group of growth factors related to muscle fibrosis, degeneration and inflammation, in a cohort of 37 symptomatic and asymptomatic AOPD patients. We compared the serum concentration of Pompe patients with a control group. We also studied whether there were differences in the serum concentration of these growth factors between symptomatic and asymptomatic patients. In parallel, we evaluated the patients using several motor function tests, spirometry, quantitative muscle MRI (qMRI), and patient-reported outcome measures (PROMs), in order to establish whether or not a correlation between serum concentration and the clinical situation of the patients exists.

## Results

### Description of the cohort

37 AOPD patients were included in the study. Twenty-nine patients were symptomatic (18 women, 62.1%) and 8 were asymptomatic. Twenty-three of the 29 symptomatic patients were already receiving ERT when first sample was obtained. In the remaining 6 symptomatic patients, blood samples were obtained before ERT was started. Asymptomatic patients were studied in neuromuscular disorder units because high levels of hepatic enzymes or CKs were found in random checkup blood analyses (5 cases) or because they had relatives already diagnosed with Pompe disease (3 cases). The demographic and clinical data of these two groups are described in Table 1. Results of the motor function tests and muscle MRI of the Pompe cohort has been already reported^15,16^. We compared the ELISA results with serums obtained from age- and sex-matched controls (n = 45).

**Table 1:** Demographic and clinical data of Pompe patients.

	Patients		
	Symptomatic	Asymptomatic	p*
Number of patients	29	8	
Gender (W)	18, 62.1%	4, 50%	0.21
Age at baseline	51 (31–65)	21 (8–51)	0.001
Time from onset of symptoms	17 (4–42)		
Patients on ERT	22	—	
Time on ERT	4.3 (1–9)	—	
Aids for walking	10	—	
Ventilation	13	—	

Comparisons between symptomatic and asymptomatic patients are shown with p-value. P was considered significant if lower than 0.05.

### Growth factor serum levels in Pompe patients compared to controls

**Figure 1:**
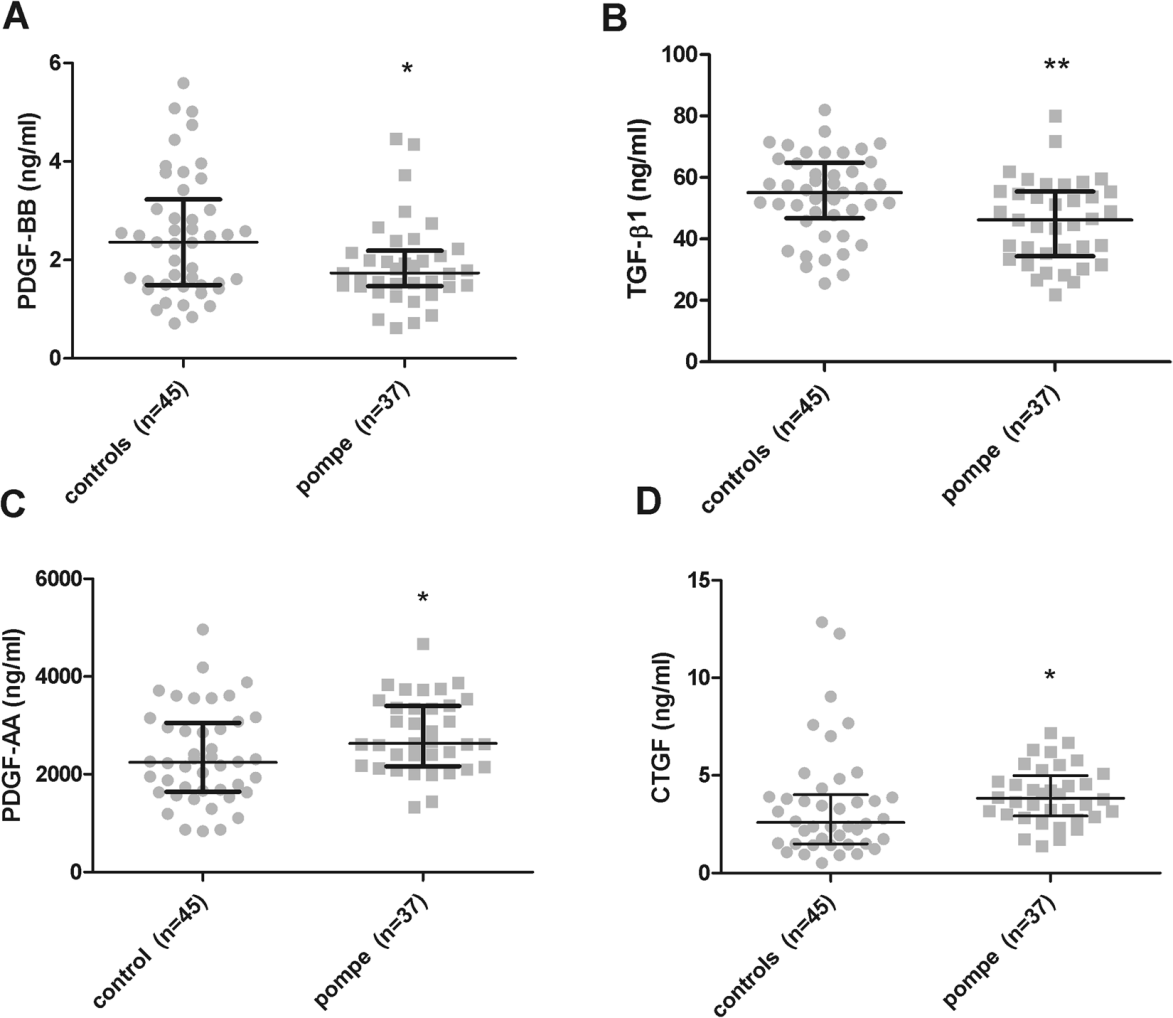
Serum levels of different cytokines in control group and pompe group. (**A**) PDGF-BB levels, (**B**) TGF-β1, (**C**) PDGF-AA and (**D**) CTGF levels were measured. Variables are represented as median and interquartile range (IQR). Statistical significance of the results by Mann-Whitney test: *p ≤ 0.05 and **p ≤ 0.01.

Our first aim was to study whether there were differences in growth factor serum levels between Pompe patients and controls. We observed significant differences in PDGF-BB, TGF-β, PDGF-AA and CTGF levels as is shown in Fig. 1. Serum PDGF-BB and TGF-β1 levels were significantly lower in Pompe patients compared to the control group. In the case of PDGF-BB, the median and interquartile range (IQR) value of Pompe serum levels were of 1.739 ng/ml (IQR: 1.467–2.186) compared to 2.360 ng/ml (IQR: 1.494–3.23) in controls (Mann-Whitney U test, p = 0.047). In the case of TGF-β1, Pompe median serum levels were of 46.17 ng/ml (IQR: 36.34–55.48) compared to 55.05 ng/ml (IQR: 46.79–64.8) in controls (Mann-Whitney U test, p = 0.007). However, serum PDGF-AA and CTGF levels were significantly higher compared to control samples. In the case of PDGF-AA, the median Pompe serum levels were 2630 ng/ml (IQR: 2161–3399) compared to 2242 ng/ml (IQR: 1642–3051) in controls (Mann-Whitney U test, p = 0.01). And in the case of CTGF, median Pompe serum levels were of 3.831 ng/ml (2.917–4.999) compared to 2.589 ng/ml (1.503–4.013) in controls (Mann-Whitney U test, p = 0.023).

### PDGF-BB levels differentiate between symptomatic and asymptomatic Pompe patients

**Figure 2:**
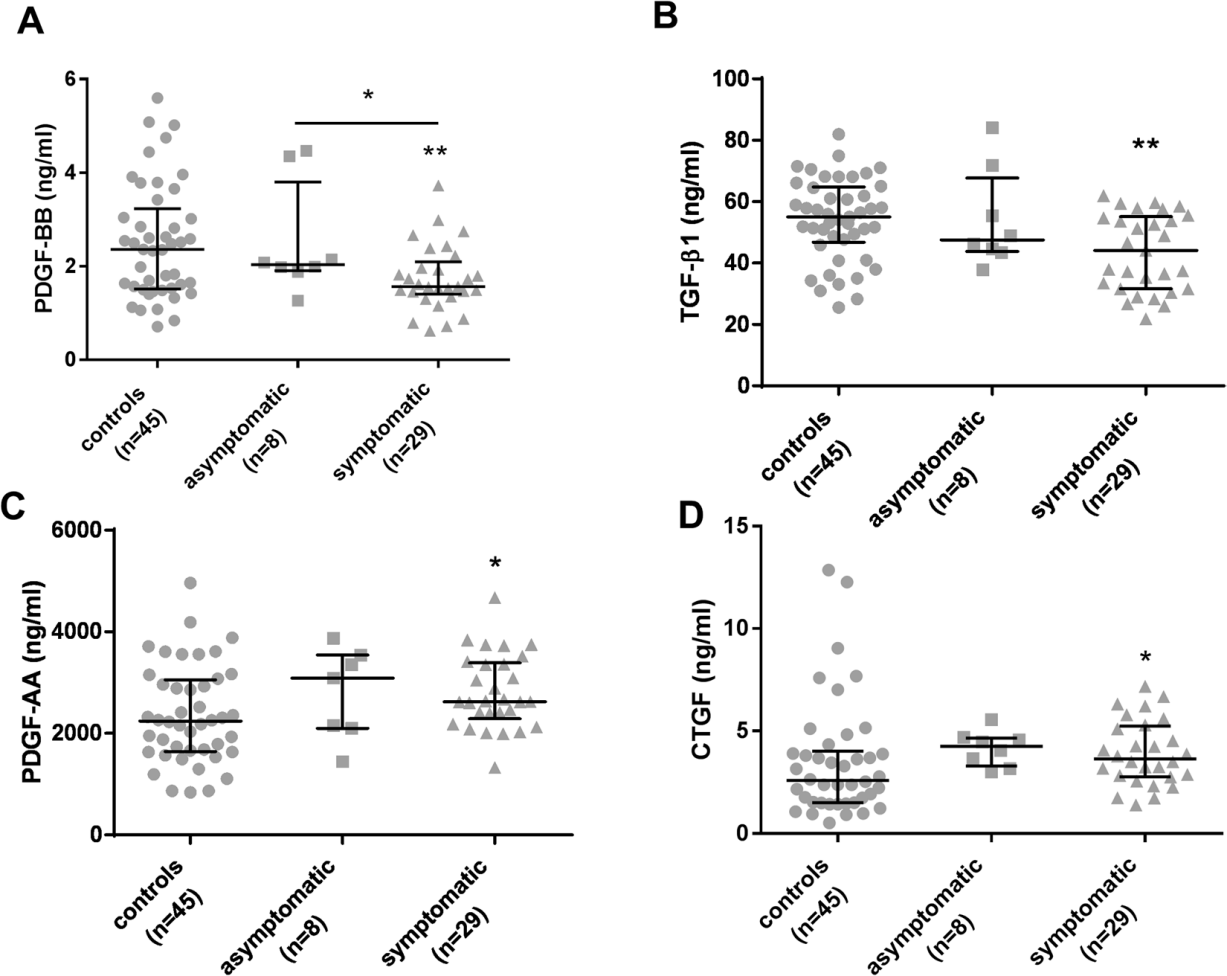
Serological levels of four growth factors in three different groups: control, symptomatic Pompe patients and asymptomatic Pompe patients. (**A**) PDGF-BB levels, (**B**) TGF-β1, (**C**) PDGF-AA and (**D**) CTGF levels were measured. Variables are represented as median and interquartile range (IQR). Comparisons were made using Mann-Whitney test. Statistical significance of the results; *p ≤ 0.05 and **p ≤ 0.01.

Our second aim was to assess whether any of the growth factors studied was able to differentiate between asymptomatic and symptomatic Pompe patients (Fig. 2). We observed that PDGF-BB levels were significantly lower in symptomatic patients (Median: 1.565 ng/ml (IQR: 1.405–2.096) compared to asymptomatic Pompe patients (Median: 2.038 ng/ml (IQR: 1.907–3.803) (Mann-Whitney U test, p = 0.044) (Fig. 2A). In contrast, we did not identify differences in TGF-β1, CTGF and PDGF-AA serum concentration between symptomatic and asymptomatic patients.

**Figure 3:**
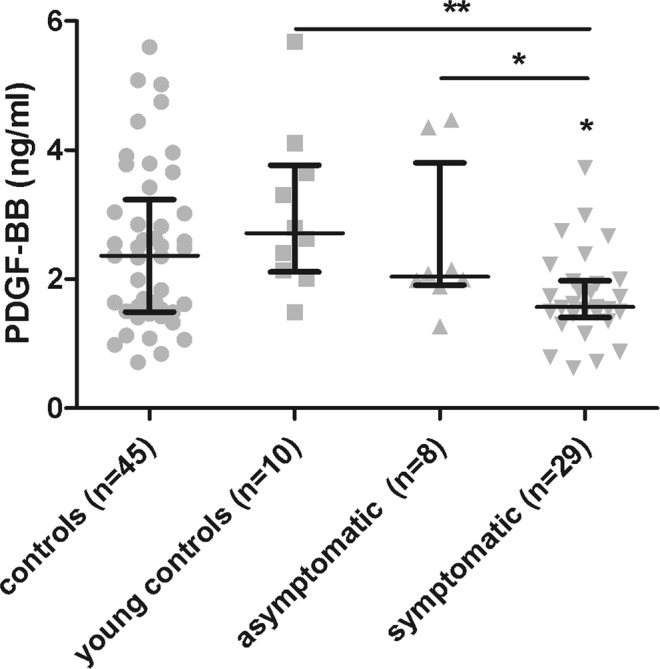
Serological levels of PDGF-BB in different groups: control, young controls, asymptomatic and symptomatic Pompe patients. Variables are represented as median and interquartile range (IQR). Comparisons were made using Mann-Whitney test. Statistical significance of the results; *p ≤ 0.05 and **p ≤ 0.01.

As there were significant differences in age between symptomatic and asymptomatic Pompe patients (Table 1), we decided to add a new group of young controls with a mean age of 23 years. We observed significant differences in PDGF-BB serum levels between symptomatic Pompe patients and all other groups, including young controls (mean age = 23 years old) (Mann-Whitney U test, p = 0.0012), all controls and asymptomatic Pompe patients. We did not observe significant differences between controls of different ages and between asymptomatic Pompe patients and young controls (Mann-Whitney U test, p > 0.05) (Fig. 3).

**Figure 4:**
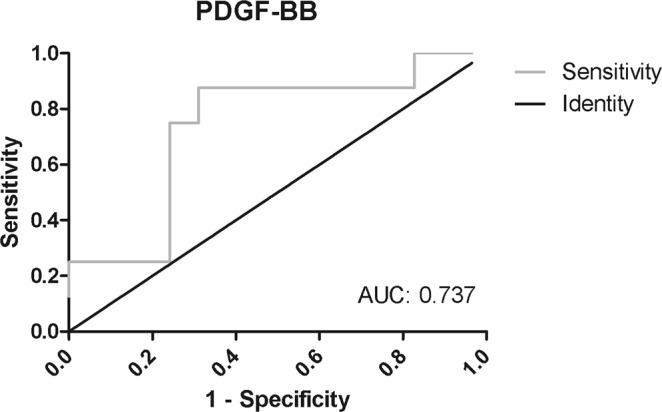
Receiver-operator characteristic (ROC) curve of the PDGF-BB for distinguishing symptomatic and asymptomatic patients. The area under the curve (AUC) for PDGF-BB was 0.737 with a P value of 0.042 and 95% confidence interval (CI) were 0.539–0.935).

To further analyze whether PDGF-BB serum levels were useful for differentiating between symptomatic or asymptomatic patients, we used a receiver operating characteristics curve, or ROC curve, and analyzed area under the curve (AUC). The ROC curve (AUC: 0.737, p = 0.042, 95%CI: 0.539–0.935) (Fig. 4) confirmed that PDGF-BB levels were able to predict which patients were symptomatic and which were asymptomatic. Therefore, patients with lower values than the cut-off level (1.97 ng/ml) had a higher probability of being asymptomatic. Sensitivity and specificity were 75% and 76% respectively.

### PDGF-BB levels decrease in Pompe patients but not in other muscle dystrophies

Since the function of PDGF-BB seems to be related with muscle regeneration, we analyzed serum levels of this growth factor in other muscle dystrophies in which regeneration increases, such as Duchenne muscle dystrophy (DMD), Becker muscle dystrophy (BMD) dysferlinopathy (DYSF) and facioscapulohumeral muscular dystrophy (FSH). Clinical and demographic features of these groups are described in Table 2.

**Table 2:** Demographic and clinical data of patients with muscle disorders included in this report.

	DMD (n:11)	BMD (n:10)	Dysferlinopathy (n:8)	FSHD (n:10)	Pompe (n:37)
Age	11.75 y.o	32.93 y.o.	35.92 y.o.	49.62 y.o.	34.5 y.o
Gender	11 men	10 men	4 men	6 men	15 men
Unassited walk	7	3	4	6	27
Aids for walking	4	3	4	4	10
Ventilator	1	1	0	1	13

DMD: Duchenne muscle dystrophy; BMD: Becker muscle dystrophy; FSHD: facioscapulohumeral muscle dystrophy; y.o.: years old.

**Figure 5:**
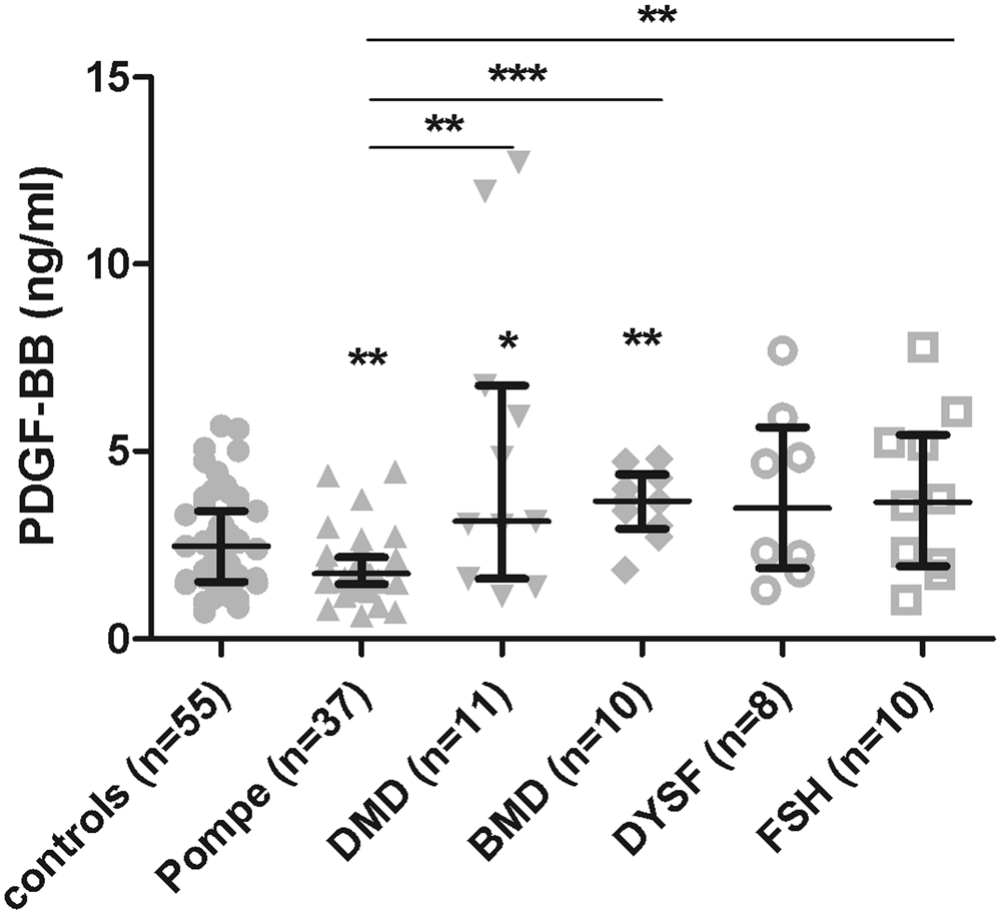
Serum levels PDGF-BB in muscle dystrophies: Pompe, Duchenne muscle dystrophy (DMD), Becker muscle dystrophy (BMD), dysferlinopathy (DYSF) and facioscapulohumeral muscular dystrophy (FSH). Variables are represented as median and interquartile range (IQR). Statistical significance of the results by Mann-Whitney test: *p ≤ 0.05, **p ≤ 0.01and ***p ≤ 0.001.

We observed that PDGF-BB levels were significantly higher in DMD (Median: 3.14 ng/ml (IQR: 1.6–6.76) (Mann-Whitney U test, p = 0.0469) and BMD (Median: 3.681 ng/ml (IQR: 2.945–4.398) (Mann-Whitney U test, p = 0.0077) compared to controls. We also obtained significant differences in DMD, BMD and FSH (Median: 3.656 ng/ml (IQR: 1.944–5.443) (Mann-Whitney U test, p = 0.0052) compared to Pompe disease (DMD: Mann-Whitney U test, p = 0.0065; DMB: Mann-Whitney U test, p = 0.0001) (Fig. 5).

### Correlation between PDGF-BB serum levels and results of muscle function tests and quantitative muscle MRI

We used the Spearman test to identify if there were any correlations between PDGF-BB serum concentration and the results of the muscle function tests, spirometry, patient-reported outcomes and qMRI. As it is shown in Table 3, we did not find any significant correlation. However, we found a non-significant tendency between PDGF-BB levels and 6MWT, the MRC score, the Myometry score, MIP, and thigh fat fraction measured using 3 point Dixon MRI.

**Table 3:** Correlation between PDGF-BB levels and results of the muscle function tests, spirometry, patients reported outcomes and qMRI.

Test	Spearman	Correlation coefficient
**Muscle function tests**		
6 MWT	*0.055*	0.33
Time to walk 10 meters	0.24	
Timed up&go test	0.81	
Time to climb 4 steps	0.17	
Time to descend 4 steps	0.26	
MRC score	*0.07*	0.31
Myometry score	*0.07*	0.31
MFM-20	0.77	
**Spirometry**		
CVF seated	1	
CVF supine	0.28	
MIP	*0.057*	−0.48
MEP	0.80	
**Patients reported outcomes**		
Activlim	0.84	
SF36	0.80	
INQoL	0.70	
**Quantitative muscle MRI**		
Thighs fat fraction	*0.08*	−0.28
Paraspinal fat fraction	0.2	

Spearman test was used to study if there was a correlation. P was considered significant if lower than 0.05. Correlation coefficient is shown for variables in which a tendency was found.

## Discussion

In the present study, we found significant differences in the serum concentration of four growth factors related to the process of skeletal muscle degeneration and regeneration in AOPD patients compared to controls. However, only serum levels of PDGF-BB were significantly different when symptomatic patients were compared with asymptomatic patients. In fact, the diagnostic accuracy of the PDGF-BB concentration to distinguish between symptomatic and asymptomatic patients was assessed by ROC curves, determining an optimal cut-off value of 1.97 ng/ml.

It is well known that chronic muscle damage leads to persistent inflammatory infiltration, muscle necrosis and activation of fibro/adipogenic progenitor (FAP) cells^17^, something that has been studied in dystrophic muscles. Eventually, muscle fibers are lost and substituted by fibro-adipose tissue^18^. Several growth factors have been related with this process, including those in the present study. TGF-β1 and PDGF-BB play an important role in satellite cell proliferation and fibrotic remodeling^19–21^. TGF-β1 is crucial in the initiation of fibrosis in skeletal muscle^20–22^, and PDGF-AA enhances the process of fibro-adipogenic expansion regulated by FAP cells^23–26^. CTGF influences the fibrotic process by inducing the expression and release of collagen type 1 by activated fibroblasts^26–28^. Although the process of muscle degeneration has been well established in animal models of muscular dystrophies such as Duchenne muscle disease, it is not yet completely known whether it happens in the same way as in Pompe disease. However, radiological studies show that skeletal muscle is gradually lost and substituted by fat tissue in patients with adult onset Pompe, mimicking what happens in patients with muscular dystrophies and suggesting a similar skeletal muscle degenerative process^29–31^. Based on this hypothesis, we decided to study the serum concentration of growth factors related with the process of muscle regeneration, degeneration and fibrosis.

PDGF-BB, which is secreted by inflammatory cells and skeletal muscle regenerative fibers, has recently been related with the process of muscle regeneration through the activation of satellite cell proliferation and chemotaxis^32^. We observed lower levels of serum PDGF-BB in AOPD patients compared to controls. Moreover, PDGF-BB serum concentration was even lower in symptomatic patients, suggesting a correlation with disease progression. As PDGF-BB probably influences muscle regeneration, the lower levels found in AOPD patients might reflect impaired regenerative response in Pompe disease, something that has also been suggested by other authors. Impaired satellite cell activation has been described in muscle samples from Pompe patients^33–35^. Moreover, serum levels of insulin growth-factor-1 and myostatin, two molecules related with the process of satellite cell activation, are lower in serum of Pompe patients compared to controls^36^.

We did not observe a significant difference in serum levels of TGF-β1, PDGF-AA and CTGF in symptomatic compared to asymptomatic Pompe patients. These three factors have been related with the process of muscles fibrosis, as discussed early. The lower levels of TGF-β1 found in Pompe patients compared to controls, supports the idea that fibrosis is not a major issue in patients with Pompe disease. In fact, Dr. Palermo^37^ and collaborators did not find an up-regulation of *TGFB1* fibrosis-associated genes in skeletal muscle Pompe patients, which supports our findings.

The lower levels of growth factors related to fibrosis and regeneration suggested by the current study could be explained by the lack of sarcolemma damage in Pompe disease. In most muscular dystrophies in which the process of muscle degeneration and regeneration has been studied, muscle damage is produced because of the instability of skeletal muscle membrane. Membrane tears induce a series of responses, such as the release by muscle fibers of cytokines that recruit inflammatory cells and participate in the activation of satellite cells. Persistent inflammatory cells release profibrotic growth factors that lead to the expansion of fibrotic tissue. The process of muscle fiber degeneration in Pompe disease is probably different. There is no evidence of necrosis or inflammatory infiltration^38^. As opposed to muscle membrane instability, lysosomal rupture has been proposed as the main mechanism leading to muscle fiber necrosis. Glycogen progressively accumulates in lysosomes producing their rupture and the release of lytic enzymes to the sarcoplasm, probably activating the process of autophagy^38,39^. It is tempting to hypothesize that local cell response is different in Pompe disease, with no recruitment of inflammatory cells, no activation of satellite cells and no release of profibrotic factors. The fact that PDGF-BB levels were higher in serum samples from patients with other muscular dystrophies, and lower in Pompe disease, supports this hypothesis.

The identification of growth factors useful for the follow-up of patients is considered one of the unmet needs in Pompe disease. ERT is being administered to those symptomatic patients, patients with muscle or respiratory muscle weakness. However, patients may develop mild motor disturbances, such as abnormal gait posture, due to the presence of mild axial involvement. Growth factors capable of differentiating between symptomatic and asymptomatic patients could then be a useful tool in follow-up. As we observed significant lower PDGF-BB levels in symptomatic Pompe patients than asymptomatic, we suggest PDGF-BB could be useful to monitorize progression of the disease and help to identify those patients in which the process of muscle degeneration has started without influencing muscle function yet. Other growth factors previously proposed, such as urine glucose tetrasaccharide Glc4 levels^40,41^, have utility in the diagnosis of Pompe disease, or in monitoring of the treatment but not for differentiating between symptomatic and asymptomatic patients^42,43^.

To summarize, we have identified a group of four growth factors related to the process of muscle degeneration and regeneration that are differently expressed in Pompe patients compared to controls. Interestingly, PDGF-BB levels were significantly different in symptomatic patients compared to asymptomatic. In our opinion, our results suggest that decreasing levels of PDGF-BB in asymptomatic patients should prompt us to tighten the follow-up of the patient, repeating muscle and respiratory function tests in order to consider starting ERT before muscle degeneration becomes irreversible.

## Methods

### Patients and study design

This study is part of an ongoing prospective open-label study in which we are following up a group of symptomatic and non-symptomatic AOPD patients annually in our center using muscle function tests, muscle MRI and blood analysis. This study has been registered in Clinicaltrials.gov (identifier NCT01914536). The present research was performed in accordance with Spanish regulation for clinical trials and studies and following the recommendations described in the Declaration of Helsinki. The study was approved by The Ethical Committee of Hospital de la Santa Creu i Sant Pau (HSCSP) in Barcelona. All participants signed an appropriate informed consent form.

The diagnosis of Pompe disease was based on the presence of two mutations in the GAA gene. In cases where a single or no mutation was detected, diagnosis was based on reduced activity in at least two tissues, lymphocytes and skeletal muscle being the most common tissues studied, as has been recently suggested by the European Pompe Consortium^10^. All patients were considered adult onset since none of them developed symptoms before the age of 18.

We defined a patient as symptomatic when we identified muscle weakness in clinical examination using the Muscle Research Council score (MRC), or when Forced Vital Capacity (FVC), while seated, was lower than 85%. A total of 37 patients were included: 23 symptomatic patients treated with ERT, 6 symptomatic patients untreated with ERT and 8 asymptomatic patients. All treated patients received 20 mg/kg acid alpha-glucosidase intravenously every other week. Untreated symptomatic patients were seen before starting ERT. Clinical and genetic features of this group of patients have been previously published^15^. In summary, mean age at baseline visit of the 23 symptomatic patients was 49.8 years old. 9 of these patients used sticks or wheelchair for walking, with two of them being fully wheelchair bound. 12 patients used non-invasive ventilation at night. Mean age of the 6 non-treated symptomatic patients was 37.4 years old. Only one patient of this group used the stick for walking and one other patient required noninvasive ventilation at night. 8 presymtomatic AOPD patients were also included (mean age 21 years, 4 women). These patients were diagnosed of Pompe disease because they were relatives of patients with Pompe or because the presence of high CK levels in blood samples. As controls we included 45 patients whose age and sex matched our Pompe cohort (mean age 48 years, 29 women), and 10 more controls whose age and sex matched with asymptomatic Pompe patients (mean age 23 years, 6 women). The controls were volunteers, most of them relatives or caregivers of our Pompe patients that kindly agreed to participate in the study. CKs levels were normal in all control patients (Reference value for our laboratory is <174 U/L).

### Growth factors identification

Blood samples were collected at baseline visit before motor function tests were performed. Blood was centrifuged for 1600 g for 9 minutes at 4 °C in order to separate the serum. The serum was aliquoted and stored at −80 °C until analysis.

Serum platelet-derived growth factor BB (PDGF-BB) and transforming growth factor β1 (TGF-β1) levels were measured using commercial enzyme-linked immunosorbent assay (ELISA) kits (R&D, Minneapolis, MN, USA), according to the manufacturer’s instructions. A platelet-derived growth factor AA (PDGF-AA) human ELISA kit was provided by ThermoFisher (Thermo Fisher Scientific, Nepean, Canada) and connective tissue growth factor (CTGF) by EIAAB Science Co (Wuhan, China). Minimum detectable cytokine concentrations for these assays were measured to be 1.7 pg/ml for TGF-β1, 15 pg/ml for PDGF-BB, 40 pg/ml for PDGF-AA and 0.18 ng/ml for CTGF. Samples were measured in duplicate and read on a microplate reader Beckman Coulter AD 340 (Beckam-Coulter, Brea, CA, USA) with AD-LD software.

### Muscle function tests

All patients were evaluated by three physiotherapists (I.B., I.P. and E.M.), with experience in neuromuscular disorders, at HSCSP in Barcelona. Physiotherapists evaluated muscle function using the following tests: the 6MWT, time to walk 10 meters, timed up-and-go test, time to climb up and down 4 steps, and the 20 item motor function measure tool (MFM-20). Muscle strength was studied using both MRC and hand-held myometry. The MRC score was the sum of all individual MRC values, while the myometry score was the sum of all individual muscle values. ACTIVLIM, INQoL and SF-36 were used as patient-reported outcome measures. Forced vital capacity while seated and in a lying position, maximum inspiratory pressure (MIP) and maximum expiratory pressure (MEP) values were obtained with a Carefusion Microlab ML 3500 MK8 spirometer (Carefusion, Yorba Linda, CA, USA).

### Muscle imaging

All patients were examined in a Philips Achieva XR 1.5 Teslas located at HSCSP. We used the same positioning protocol for all patients: supine position with legs stretched, the patella facing upward and the ankles in a neutral position.

3D 3-point Dixon images were acquired with the following acquisition parameters: TR/TE = 5.78/1.8, 4 ms, flip angle = 15°, FOV = 520 × 340 × 300 mm, voxel size = 1 × 1 × 3 mm.

Analysis of the 3-point Dixon MR images was performed using the PRIDE (Philips Research Image Development Environment) tool, as has been reported previously^15,42^. ROIs were manually drawn on five slices of the following muscles: *rectus femoris*, *vastus intermedius*, *vastus lateralis*, *vastus medialis*, *adductor magnus*, *sartorius*, *gracilis*, *semitendinosus* and *semimembranosus*, and on three slices of *biceps femoris long head*, *biceps femoris short head* and *adductor longus*

### Data analysis

Non-parametric tests were used for the statistical analysis of the variables. The Mann-Whitney U test investigated whether there were significant differences in variables between groups (symptomatic vs asymptomatic and control vs Pompe). We used Spearman’s rank correlation (coefficient reported as ρ) to investigate any correlation between the serum concentration of growth factors and the results of the muscle function tests, spirometry, quality of life scales and the thigh fat fraction obtained using qMRI. As we ran multiple correlations, a Bonferroni test was performed to avoid type 1 errors. Finally, a ROC curve was performed to study whether PDGF-BB levels were able to differentiate between symptomatic and asymptomatic Pompe patients with high sensitivity and specificity. The results of all statistical studies were considered significant if P was lower than 0.05. Statistical studies were performed using IBM SPSS® Statistics software version 21. The datasets generated during the current study are available from the corresponding author on reasonable request.

## Data Availability

The datasets generated during and/or analysed during the current study are available from the corresponding author on reasonable request.
